# Vanadium Toxicity Is Altered by Global Warming Conditions in Sea Urchin Embryos: Metal Bioaccumulation, Cell Stress Response and Apoptosis

**DOI:** 10.3390/jox14030064

**Published:** 2024-08-22

**Authors:** Chiara Martino, Fabiana Geraci, Rosaria Scudiero, Giampaolo Barone, Flores Naselli, Roberto Chiarelli

**Affiliations:** 1 Department of Biological, Chemical and Pharmaceutical Sciences and Technologies (STEBICEF), University of Palermo, Viale delle Scienze, 90128 Palermo, Italy; chiara.martino@unipa.it (C.M.); fabiana.geraci@unipa.it (F.G.); giampaolo.barone@unipa.it (G.B.); flores.naselli@unipa.it (F.N.); 2 National Biodiversity Future Center, Piazza Marina 61, 90133 Palermo, Italy; 3 Department of Biology, University Federico II, 80126 Napoli, Italy; rosaria.scudiero@unina.it

**Keywords:** climate change, marine ecotoxicology, heat shock proteins, DNA fragmentation, toxicity biomarkers

## Abstract

In recent decades, the global vanadium (V) industry has been steadily growing, together with interest in the potential use of V compounds as therapeutics, leading to V release in the marine environment and making it an emerging pollutant. Since climate change can amplify the sensitivity of marine organisms already facing chemical contamination in coastal areas, here, for the first time, we investigated the combined impact of V and global warming conditions on the development of *Paracentrotus lividus* sea urchin embryos. Embryo-larval bioassays were carried out in embryos exposed for 24 and 48 h to sodium orthovanadate (Na_3_VO_4_) under conditions of near-future ocean warming projections (+3 °C, 21 °C) and of extreme warming at present-day marine heatwave conditions (+6 °C, 24 °C), compared to the control temperature (18 °C). We found that the concomitant exposure to V and higher temperature caused an increased percentage of malformations, impaired skeleton growth, the induction of heat shock protein (HSP)-mediated cell stress response and the activation of apoptosis. We also found a time- and temperature-dependent increase in V bioaccumulation, with a concomitant reduction in intracellular calcium ions (Ca^2+^). This work demonstrates that embryos’ sensitivity to V pollution is increased under global warming conditions, highlighting the need for studies on multiple stressors.

## 1. Introduction

Marine ecosystems constantly receive as much as 80% of anthropogenic contaminants from terrestrial sources. Therefore, monitoring chemical pollution in the marine environment and analyzing the effects on the marine biota is becoming a key target for environmental management [1]. Vanadium (V) is a ubiquitous trace metal on Earth, used in different manufacturing and industrial processes, mainly located in coastal ecosystems, thus leading to its release into the marine environment [2]. V consumption worldwide doubled between 2002 and 2013 [3], reaching 109,700 tons in 2020 [4], and leading to a concentration in seawater ranging from 2.4 µg L^−1^ in Oceania to 120 μg L^−1^ in Asia [5], causing a potential threat to marine species. However, despite being classified as a potentially toxic metal by the United States Environment Protection Agency (USEPA) [6], V has been given limited attention in comparison to similar trace metals of emerging concern [5].

At the same time, ocean warming is posing serious threats to marine ecosystems. While over the past 40 years the global warming of sea surfaces recorded an increase of 0.15 °C per decade, a further 3.5 °C increase is predicted in a non-mitigated scenario by 2099 [7]. Extreme temperatures have been documented during marine heatwaves, with regional average increases of 6 °C or more, causing episodes of mass mortalities from the Mediterranean Sea to the tropics [8,9,10], and thus representing an urgent risk [7,11]. The Mediterranean Sea is considered a hotspot of climate change, with a projected 20% higher increase in seawater temperature than in global oceans [12]. Warming and marine heatwaves resulted in five consecutive years (2015–2019) of widespread mass mortality of Mediterranean marine species [10].

While it is known that chemical pollution and changes in temperature can individually affect the health status of marine species, exposure to their mixture might cause negative synergistic consequences with underestimated effects [13]. Therefore, one of the current challenges is to apprehend how climate change will alter the impact of pollution on marine organisms, analyzing the responses of local species. Changes in seawater temperature could alter metals’ toxicity, modifying their bioavailability and their skill to act as bioactive metabolites [14]. Since increased temperatures may enhance the bioavailability of metals due to higher solubility and increased ventilation and feeding activity, caused by greater energy demand [15,16,17,18], ocean warming conditions could increase metal bioaccumulation in marine organisms.

The benthic and planktonic larvae of different marine species living in the Mediterranean coastal regions are exposed to combined increasing temperatures and pollution and are considered susceptible developmental life stages [13,14,19]. *Paracentrotus lividus* sea urchin embryos and larvae are commonly used to assess environmental quality, being highly sensitive to pollution and climate change [19,20,21]. Their larval skeleton is a sensitive tool in ecotoxicological and global change studies, since the genetic regulatory network as well as the cellular/molecular mechanisms underlying its growth have been extensively revealed [20,22,23]. Biomineralization involves the absorption of calcium and magnesium present in seawater, starting from the gastrula stage [24], to construct the complex calcite (CaCO_3_) endoskeleton that characterizes the pluteus stage [20]. Due to their ecological influence, understanding the impacts of concomitant pollution and warming on *P. lividus* development is crucial to recognize this species’ vulnerabilities and resilience for management and conservation.

Single stressor studies have explored the effects of metal ion pollution (e.g., cadmium, manganese, gadolinium) on *P. lividus* embryos, showing growth impairment and skeletal malformations [25,26,27]. Using a wide range of sublethal V concentrations (50 nM–1 mM) on *P. lividus* embryos, we have previously shown the induction of severe malformations, mainly affecting skeleton formation, the activation of the HSP-mediated stress response, the induction of autophagy and apoptosis and V bioaccumulation in a dose- and time-dependent manner [28,29]. On the other hand, our previous study testing temperatures across near-future projections (+3 °C, 21 °C) and present-day heatwave conditions (+6 °C, 24 °C) showed that *P. lividus* development and skeletal growth has a thermal threshold, with high survival and normal development/biomineralization at 21 °C and the induction of developmental abnormalities at 24 °C [14]. Larvae exposed to increased temperatures also showed a greater level of fragmented chromatin, an indicator of apoptosis activation [14].

Only a few multistressor studies analyzed the concomitant exposure to chemical pollutants and climate change conditions in *P. lividus* embryos. Recent research indicated that ocean warming and acidification will increase their sensitivity to chemical pollutants, such as chlorpyrifos and microplastics [13]. Another study showed that, while gadolinium (Gd) pollution at the physiological temperature (18 °C) caused a stunting effect and impaired skeleton growth [30], a concomitant +3 °C increase (21 °C) reduced the negative effects of Gd on development, while a +6 °C increase (24 °C) resulted in negative synergistic effects [14].

In this paper, for the first time, we present a multistressor study of the combined effects of V pollution and increased temperatures on the development of a marine species, the sea urchin *P. lividus*. Our aim is to investigate how moderate warming (21 °C) and marine heatwave conditions (24 °C) might alter the effects of 1 mM V embryo exposure. We quantified the bioaccumulation of V and Ca in response to V and increased temperatures and analyzed the morphological, cellular and molecular responses in embryos and larvae, studying the effects on embryo development and the induction of defense mechanisms, including HSP production and apoptosis.

The chosen V concentration is the lowest dose, causing 100% developmental abnormalities in *P. lividus* embryos, in terms of phenotypic plasticity and the modulation of the response strategies, for which we have previously fully characterized the toxicological mechanisms and the molecular responses activated [29]. In the present work, this dose was chosen to analyze the defensome of *P. lividus* as a bioindicator organism to help us understand which molecular strategies can make species more resilient to the combined effects of climate change and pollution.

Characterizing the impact of anthropogenic pollution under a climate warming scenario can improve our understanding of responses of local marine species, which could serve to provide potential implications to decision makers for environmental management.

## 2. Materials and Methods

### 2.1. Embryos Cultures, Treatments, and Morphological Analyses

Eggs and sperms were obtained from adult *P. lividus* sea urchins collected from the north-western coasts of Sicily, as previously reported [14]. In vitro fertilization was carried out using 4000 eggs/mL and 10 µL of dried sperm diluted in 1 mL of Millipore filtered sea water (MFSW) to promote sperm activation. Before fertilization, sperm maturity (mobile spermatozoa) and egg quality (spherical morphology) were microscopically inspected. After fertilization, the embryos were maintained in glass containers with gentle mixing, as described by the authors of [31].

Three aliquots of embryo cultures were grown at three different temperatures: physiological (18 °C), near-future projections (+3 °C, 21 °C) and present-day heatwave conditions (+6 °C, 24 °C). Simultaneously, three aliquots of embryo cultures developed under the same temperatures were exposed to 1 mM sodium orthovanadate (Na_3_VO_4_, thereafter V) (Sigma-Aldrich, S6508, Waltham, MA, USA), as described by Chiarelli et al. [29]. Embryo development was performed using three thermostatic chambers (Angelantoni Scientifica, Massa Martana, PG, Italy) equipped with a rotating propeller (25 rpm). Temperatures were automatically regulated using temperature sensors in the chambers and were found to be stable (±0.3 °C). Microscopic analyses of the six treatments were performed after 24 and 48 h post fertilization using a digital camera (Nikon Sight DS-U1, Tokyo, Japan) connected to an Olympus BX50 microscope (Olympus Corporation, Tokyo, Japan), under a 10X objective. About 100 embryos for each condition were analyzed and classified according to the criteria previously defined [28].

### 2.2. V and Ca Inductively Coupled Plasma Mass Spectrometry Analyses

The incorporated V and Ca contents in embryo cells were determined by Inductively Coupled Plasma Mass Spectrometry (ICP-MS), as reported elsewhere [29]. Metal quantification was carried out in about 250,000 embryos and expressed as the mean ± standard deviation (SD) of triplicate experiments (*n* = 3). The correspondence of nominal vs. analytical V concentrations was tested in the initial embryonic culture medium (0 h), at 24 and 48 h, as previously reported [29]. The Limit of Quantification (LOQ) was 4.5 µg/L for V and 500 µg/L for Ca, while the Limit of Detection (LOD) was 1.8 µg/L for V and 200 µg/L for Ca. For both elements, the certified reference material was >95%.

### 2.3. Immunoblot Detection

Electrophoretic and immunoblotting analyses were performed according to previous work from our team [25]. The following antibodies were used: mouse monoclonal anti-heat shock protein 60 (HSP 60) clone LK2 (Sigma-Aldrich, H3524); rabbit polyclonal anti-cleaved caspase-7 (Asp198) (Cell Signaling Technology, 9491, Danvers, MA, USA); and rabbit polyclonal anti-actin (20–33) (Sigma-Aldrich, A5060), diluted, respectively as follows: 1:500, 1:750 and 1:500. As secondary antibodies, anti-rabbit and anti-mouse IgG horseradish peroxidase linked whole antibodies, diluted to 1:2500 with TBS-T, were used. Actin band intensity was used as the loading control. The data are presented as means ± standard deviations (SDs) of triplicate experiments (*n* = 3).

### 2.4. Terminal Deoxynucleotidyl Transferase (TdT) dUTP Nick-End Labeling (TUNEL) Assay

Nuclei containing fragmented chromatin were highlighted by a TUNEL assay (Invitrogen, A23210, Waltham, MA, USA) through an in situ analysis on whole-mount embryos, as described by the authors of [29]. The fragmented DNA nuclei were observed under a 20X objective of a fluorescence microscope (Olympus BX50, Tokyo, Japan).

### 2.5. Statistical Analysis

Statistical analysis was performed as described by Martino et al. [14]. All the data were analyzed using Statistica 13.2 software (StatSoft, Tulsa, OK, USA), with *p* < 0.05 as the level of significance. A two-way analysis of variance (ANOVA) was carried out with V concentration and temperature as fixed factors.

## 3. Results

### 3.1. Temperature Increase Alters the Effects of V on Embryo Development

**Figure 1:**
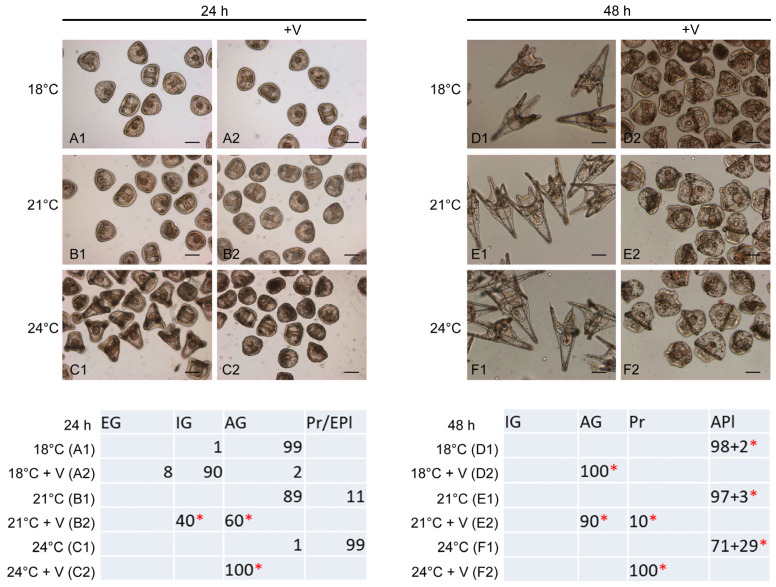
Morphological analysis of *P. lividus* embryos. Upper panel: images of representative embryos and larvae at 24 and 48 h of development. Embryos were grown at three different temperatures (18 °C: (**A1**,**A2**,**D1**,**D2**)); 21 °C: (**B1**,**B2**,**E1**,**E2**); and 24 °C: (**C1**,**C2**,**F1**,**F2**)) in the presence (**A2**–**F2**) or absence (**A1**–**F1**) of V. Bar: 100 μm. Lower panel: Number of embryonic stages detected in all treatments. Developmental stages are indicated as EG (early gastrula); IG (intermediate gastrula); AG (advanced gastrula); Pr (prism); EPl (early pluteus); and APl (advanced pluteus). * Altered phenotypes. Data are presented as means of triplicate experiments (*n* = 3).

Both V (F_5,12_ = 970.7, *p* < 0.0001) and temperature (F_5,12_ = 567.3, *p* < 0.0001), as well as their interaction (ANOVA V x temp: F_5,12_ = 507.7, *p* < 0.0001), had a significant effect on *P. lividus* development (Figure 1(A1–F2)). Control embryos grown at 18 °C, 24 h post fertilization, were advanced gastrulae (AG), characterized by an adequate archenteron extension and triradiate spicules formation (Figure 1(A1)), and advanced plutei (Apl) after 48 h, with a well-developed, symmetrical larval skeleton. More advanced stages occurred as temperatures increased (Figure 1(E1–F1)), confirming the positive correlation between warming and increased rate of development, as previously stated [14]. However, 29% of larvae grown at 24 °C were abnormal plutei.

On the contrary, 98% of V-exposed embryos grown at 18 °C for 24 h showed a general delay in development with no malformations, being 90% at the IG stage and 8% at the EG stage, while only 2% were at the AG stage (Figure 1(A2)), confirming our previously obtained results [28].

Strikingly, 100% of V-exposed embryos incubated at 21 and 24 °C showed morphological malformations (Figure 1(B2–C2)). Specifically, V-exposed embryos at 21 °C displayed anomalies concerning imperfect symmetry and an incorrect distribution of cells forming the archenteron (Figure 1(B2)), with 60% of embryos at the AG stage and 40% at the IG stage. V-exposed embryos at 24 °C were all at the AG stage, with evident morphological anomalies concerning the symmetry and cell distribution of all tissue districts throughout the embryo (Figure 1(C2)).

After 48 h of development, 100% of V-exposed embryos at 18 °C were malformed AG, showing an absence of the skeleton and an altered distribution of the cells of various embryonic districts (Figure 1(D2)). The increase in temperatures caused 10% (21 °C) and 100% (24 °C) of V-exposed embryos to be at an advanced stage (Pr stage) compared to treated embryos grown at 18 °C (Figure 1(D2–F2), respectively), with evident anomalies related to the absence of a skeleton, altered symmetry and irregular cell distribution (Figure 1(E1,E2,F2)).

### 3.2. V and Ca Content Are Modulated by Temperature Increase

**Figure 2:**
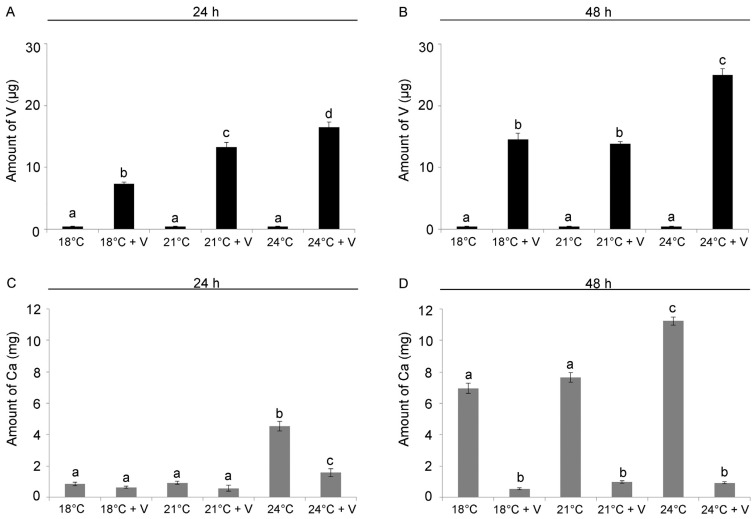
Histograms showing quantitative analysis of the amount of V (**A**,**B**) and Ca (**C**,**D**) incorporated after 24 and 48 h of development/treatment. Embryos were cultured in V 1 mM at 18, 21 and 24 °C. V and Ca contents were detected by Inductively Coupled Plasma Mass Spectrometry (ICP-MS), determining mean metal quantity in about 250,000 embryos. Data are presented as means ± standard deviations (SDs) of triplicate experiments (*n* = 3). Treatments with same letter do not differ (Tukey HSD).

We used ICP-MS to determine the influence of temperature on V bioaccumulation and on the intracellular Ca content. At 24 h of development/treatment, both V (F_5,12_ = 310.8, *p* < 0.0001) and temperature (F_5,12_ = 111.2, *p* < 0.0001) significantly affected V bioaccumulation, as well as their interaction (V × temp: F_5,12_ = 78.3; *p* < 0.0001). We detected a temperature-dependent increase in the amount of V bioaccumulated in embryos (Tukey’s HSD: 18 °C < 21 °C < 24 °C), with a 50% and 60% increase at 21 and 24 °C, respectively, if compared to 18 °C. As expected, no V was detected in embryos grown without the addition of the metal (Figure 2A).

At 48 h of development/treatment, V (F_5,12_ = 974.6, *p* < 0.0001), temperature (F_5,12_ = 161.3, *p* < 0.0001) and their interaction (V × temp: F_5,12_ = 57.9; *p* < 0.0001) significantly affected V bioaccumulation. V-exposed embryos in the 24 °C treatment showed a 40% increase in the amount of V if compared to embryos reared at 18 °C, while no difference was detected for embryos grown at 21 °C (Tukey’s HSD: 18 °C = 21 °C < 24 °C). Again, as expected, no V was found in embryos grown without V (Figure 2B).

After 24 h of development, V-exposure (F_5,12_ = 169.7, *p* < 0.0001) and temperature (F_5,12_ = 290.8, *p* < 0.0001), as well as their interaction (V × temp: F_5,12_ = 97.8; *p* < 0.0001), significantly affected Ca accumulation as well. No significant difference in the quantity of intracellular Ca was observed between embryos grown at 18 °C and 21 °C, both with and without V. Interestingly, embryos grown at 24 °C had an 80% greater amount of Ca than embryos grown at lower temperatures (18 °C and 21 °C) and 67% more than embryos in 24 °C + in the presence of V, showing that the increase in temperature was the main driver for Ca bioaccumulation at 24 h (Figure 2C).

At 48 h, Ca accumulation was affected by V-exposure (F_5,12_ = 977.9, *p* < 0.0001), temperature (F_5,12_ = 187.8, *p* < 0.0001) and their interaction (V × temp: F_5,12_ = 150.1, *p* < 0.0001). While V-exposed larvae presented low amounts of Ca at all temperatures, a 36% increase was found at 24 °C if compared to 18 °C (Tukey’s HSD: 18 °C + V = 21 °C + V = 24 °C + V < 18 °C = 21 °C < 24 °C, Figure 2D).

### 3.3. Embryonic Cytoprotection and Apoptosis

**Figure 3:**
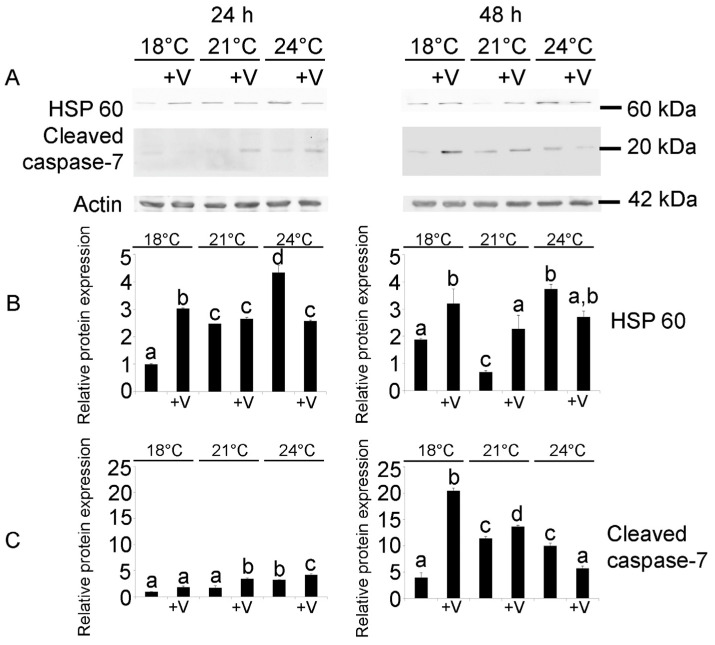
Immunoreactive bands obtained by immunoblotting detection and quantitative analysis for HSP 60 and cleaved caspase-7. (**A**) Total lysates of control and treated embryos after 24 and 48 h of development/treatment. Actin was used as loading control. Histograms show densitometric analysis of bands identified for (**B**) HSP 60 and (**C**) cleaved caspase-7. Relative protein expression, reported as arbitrary units, was calculated as band density ratio to that of actin. Data are presented as means ± standard deviations (SD) of triplicate experiments (*n* = 3). Data were analyzed by two-way ANOVA. Treatments with same letter do not differ (Tukey HSD).

At 24 h, V had a significant effect on HSP60 expression (F_5,12_ = 149.1; *p* < 0.0001), as well as temperature (F_5,12_ = 475.8; *p* < 0.0001) and their interaction (temp × V: F_5,12_ = 791; *p* < 0.0001). V-exposed embryos at 18 °C showed a 57% increase in HSP 60 expression compared to the 18 °C controls, while at increased temperatures, warming was the main driver of HSP 60 induction, with a 60% and 78% increase at 21 and 24 °C, respectively (Figure 3), in accordance with our previous results [14]. This finding suggests that embryos simultaneously exposed to V and increased temperatures may activate other strategies to cope with the stress.

Similarly, at 48 h, the HSP 60 protein levels were significantly affected by V (F_5,12_ = 35.29; *p* < 0.0001), temperature (F_5,12_ = 14.27; *p* = 0.002) and their interaction (temp × V: F_5,12_ = 16.8; *p* < 0.0005). Larvae grown at 24 °C showed a 50% increase with respect to 18 °C, while no difference was present at 21 °C, probably because larvae at this temperature were able to restore an optimal state and to respond effectively to the stress induced by this modest increase in temperature (Figure 3A,B). V-exposed larvae, both at 18 °C and 21 °C, had a 20% and 70% increase if compared to larvae reared at the same temperatures without V.

At both developmental endpoints (24 and 48 h), the embryos exposed to V at 24 °C showed lower levels of HSP 60 compared to the corresponding embryos reared without V, suggesting the activation of other defense mechanisms.

The effects of V treatment and temperature increase on caspase-7, one of the effector caspases of apoptosis, were also analyzed. At 24 h, the protein levels of cleaved caspase-7 were barely detectable by WB (Figure 3A,C), with no effect of both temperature and V on its expression, as was already shown for embryos exposed to Gd and increased temperatures [14].

On the contrary, at 48 h, the increased amount of the cleaved caspase-7 protein showed that V (F_5,12_ = 526.2; *p* < 0.0001) had a significant effect, as well as temperature (F_5,12_ = 227.9; *p* < 0.0001) and their interaction (temp × V: F_5,12_ = 872.19; *p* < 0.0001). Larvae exposed to higher temperatures had a 60% increase in the cleaved caspase-7 levels compared to larvae cultured at 18 °C (Figure 3A,C). With respect to the effect of V on cleaved caspase-7 protein expression, at 18 °C, V-exposed larvae had an 80% increase compared to the controls, while only a slight modulation was observed at 21 and 24 °C between larvae developed with or without V.

### 3.4. DNA Fragmentation

Since the presence of cleaved caspase-7 is a clear sign of the execution of the apoptotic pathway, we conducted an in situ TUNEL analysis on whole embryos to confirm the activation of apoptosis and to highlight the number and the type of the cells affected.

**Figure 4:**
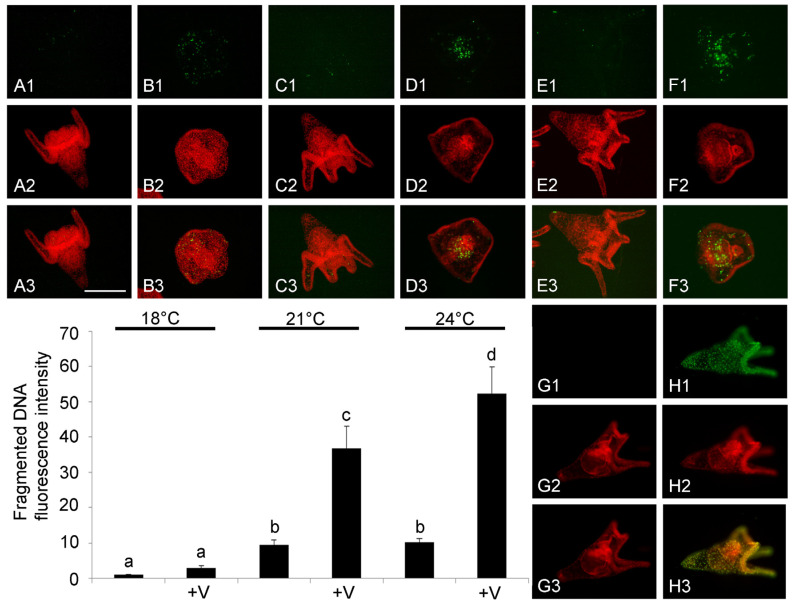
Apoptotic nuclei evalutation in whole-mount embryos detected by fluorescent TUNEL assay and densitometric analysis. Pictures of demonstrative embryos at 48 h of development/treatment. DNA fragmentation (**A1**–**H1**). Nuclei marked with propidium iodide (**A2**–**H2**). Merging of both signals (**A3**–**H3**). Control embryo reared at 18 °C (**A1**–**A3**); V-treated embryo at 18 °C (**B1**–**B3**); embryo grown at 21 °C (**C1**–C3); V-treated embryo at 21 °C (**D1**–**D3**); embryo grown at 24 °C (**E1**–**E3**); and V-treated embryo at 24 °C (**F1**–**F3**). Negative control embryo (**G1**–**G3**). Positive control embryo (**H1**–**H3**). Bar = 100 μm. Data in histograms report relative quantification of green fluorescence related to fragmented DNA as mean relative levels expressed in arbitrary units as fold change compared to 18 °C value, assumed as 1 in histogram, and are presented as means ± standard deviations (SDs) of triplicate experiments (*n* = 3) of entire morphological population. Data were analyzed by two-way ANOVA. Treatments with same letter do not differ (Tukey HSD).

V-exposure (F_5,48_ = 463.3, *p* < 0.0001), temperature (F_5,48_ = 250.2, *p* < 0.0001) and their interaction (F_5,48_ = 112.8, *p* < 0.0001) significantly influenced the levels of fragmented DNA at 48 h. Larvae exposed to both increased temperatures had a 10-fold increase in the levels of fragmented DNA if compared to larvae reared at 18 °C, confirming our previous results [14]. Tukey’s HSD test showed no difference between V-exposed larvae and controls at 18 °C, while a significant four- and five-fold increase was, respectively, observed at 21 and 24 °C in V-exposed larvae compared to larvae cultured without V (Figure 4).

The TUNEL fluorescence assay allowed us to localize the apoptotic nuclei with fragmented DNA, showing a peculiar arrangement. The presence of fragmented nuclei during normal development appears to be associated with physiological apoptosis, and mainly concerns some cells arranged along the apical-, pre- and post-oral arms (Figure 4(A1)). This arrangement is also found in embryos exposed to increased temperatures (21 and 24 °C), although it involves a greater number of cells (Figure 4(C1,E1)).

In V-exposed larvae in the 21 °C treatment, nuclei with fragmented DNA were mainly distributed in the primitive intestine during the convergent extension phase (Figure 4(D1)), while in V-exposed embryos reared at 24 °C, the nuclei with fragmented DNA were not only limited to the intestine but were also present in other embryo tissues (Figure 4(F1)). Overall, this analysis showed that V-exposure and increased temperature had a negative synergistic effect on the occurrence of apoptotic nuclei with fragmented DNA.

## 4. Discussion

The present study investigated the concomitant effects of V pollution and seawater warming conditions on the development of *P. lividus* embryos and larvae. We analyzed phenotypic plasticity and the activation of cellular defense mechanisms within the embryo defensome, in a climate change perspective, represented by two realistic warming conditions: moderate warming, as predicted in a non-mitigated scenario, by 2099 (21 °C) and current marine heatwave conditions (24 °C) [7]. Recent research highlighting the global threat of V compounds in marine ecosystems indicated invertebrates (e.g., crustaceans, echinoderms, mollusks) as the most sensitive animals to V [5]. Since there is a lot of interest in the possible pharmacological role of this element, in its various oxidation states and different coordination number and geometry, and studies on its safety profile are lacking, further investigation in various animal models is needed before starting a clinical trial [32]. The sea urchin embryo represents an interesting bioindicator model system that offers the opportunity to detect several environmental changes both from a morphological and a molecular point of view [20,33].

At a morphological level, we found that increased temperature accelerated embryo development, probably due to an increased metabolism [34]. However, under marine heatwave conditions (+6 °C, 24 °C), we also found an increased percentage (30%) of embryos with abnormal morphology, confirming our previously obtained results [14]. Thus, exceeding the thermotolerance threshold of *P. lividus* led to severe morphological alterations, accompanied by an increased HSP 60 expression and apoptosis activation. This aspect shows how embryos under severe stress conditions try to establish new adaptive phenotypes in order to safeguard the development program.

Using a multistressor approach, we assessed the combined effects of V-exposure and increased temperature. Our results show how the machinery for the toxicological response to V is modulated by increased temperatures. V-exposure at the physiological temperature (18 °C) caused a delay in development after 24 h, and a stunting effect on larvae development and on skeleton formation at 48 h, as previously reported [28]. In combination with increased temperature, we observed a negative synergistic effect, with 100% of abnormal phenotypes both at 24 and 48 h.

V bioaccumulation positively correlated with temperature increase, as there was an increased level of the metal in embryos grown at 21 and 24 °C. This trend could be due to an enhanced solubility and kinetic of V at higher temperatures, and/or to an enhanced activity of free metal ions, making them more bioavailable [15,16,17,18]. High temperatures are known to improve ingestion speed and to increase the accumulation of pollutants in the body, while detoxification mechanisms could differ depending on the life stage [35]. Warming also increased the bioaccumulation of calcium ions in *P. lividus* embryos, while V exposure inhibited its uptake. We can speculate that the impairment of spicule formation in V-exposed embryos might be caused by the action of V ion as a Ca channel blocker through a competitive effect, as has been proposed for other metals that disrupt skeleton formation in *P. lividus* embryos [26,27,36]. Since arm length is responsible for the swimming and the feeding ability of sea urchin larvae, and therefore ultimately for their developmental success [23,37], it is likely that V exposure would reduce *P. lividus* embryo survival in nature.

It is known that marine organisms are able to handle thermal injury to proteins, nucleic acids and membranes using a graded cellular stress response that is modulated by the degree of cellular damage [38]. Heat stress denatures proteins and induces a rapid transcription of genes encoding HSPs to help re-establish the native structures of thermally unfolded proteins [38]. Severe thermal stress may cause the breakage of DNA, whose extent may exceed capacities of molecular repair, leading to additional adaptive responses, such as the removal of entire cells through programmed cell death (apoptosis) [38,39].

Our previous studies demonstrated that *P. lividus* embryos possess a hierarchical and finely orchestrated response to environmental stressors. HSP synthesis is the first line of defense, initiated soon after exposure to warming conditions [14] or to other stressors, including chemical contaminants [20]. While a low level of basal physiological apoptosis is essential for proper development [40], depending on the extent of the damage, selective apoptosis is activated to eliminate the most impaired cells, but, if the damage is too extensive, massive apoptosis is induced throughout the embryo, leading to its death [41].

Exposure to chemical pollutants, such as Gd and V, as well as the exposure to natural marine toxins [42], are known to activate cell-selective apoptosis in *P. lividus* embryos [29,42]. These processes, being expressed in live embryos, fluctuate over time, continuously acting on the variation in protein concentration, promoting degradation [21] and subtraction for cell loss [25,43]. Consequently, the level of protein markers of these processes could change drastically within a few minutes or even within the same time lapse. The possibility of studying the interplay between these mechanisms on the whole embryo allows us to obtain information on the stress response at the organism level, where the communication network between cells is preserved.

The present study confirmed that, at 24 and 48 h, the increase in temperature promoted the activation of defense strategies against thermal stress, such as the induction of HSP 60. On the contrary, at 48 h, we observed the activation of cell-selective apoptosis in a subset of damaged cells as a last defense strategy to protect the development program.

Interestingly, the diversification of alternative developmental phenotypes is also confirmed by the localization of the cells with fragmented DNA. While embryos grown at 18 °C had few apoptotic cells with a homogeneous distribution throughout the embryo, embryos exposed to increased temperatures displayed a diversification of the tissue districts involved, demonstrating how embryos can alter their specific response in response to global warming conditions.

Overall, this work demonstrates that global warming conditions enhance the sensitivity of *P. lividus* embryos to V pollution. This climate change perspective indicates that global warming conditions will have a synergistic negative effect with respect to V pollutions, as was already shown for other pollutants [13,14]. The gradual warming scenario (IPCC projections for 2100, +3 °C) would possibly provide the potential for species adaptation/acclimatization, while extreme heatwave events (+6 °C) give far less scope for acclimatization. Our findings indicate that climate change conditions will have a serious impact on the marine biota, causing negative synergistic effects with toxic pollutants already present in the sea.

## 5. Conclusions

Wildlife populations and their habitats are currently exposed to an increasing amount and variety of stressors caused by human activities, together with the increasing pressure of climate change [44]. Interaction between global warming and environmental pollution represents a worrying factor, as it induces unpredictable effects on living organisms. For this reason, scientific research should deepen our understanding of the toxic effects induced by persistent pollutants in the marine environment through a climate change perspective for informing ecosystem management.

The sea urchin embryo, being very sensitive to variations in the environment, represents a bioindicator that could provide valuable and predictive information on climate change. This study highlighted the effects of V pollution under ocean warming conditions, demonstrating that the morphological and cellular responses of sea urchin larvae vary greatly when individually exposed to each stressor (V pollution or temperature increase) from the moment of exposure to their combined effect. Our findings support the concept that combined effects cannot be reliably predicted from the individual effects of each stressor, and emphasize the need to employ holistic approaches to understand how stressors interact to provide useful predictions to management.

## Data Availability

The data that support the findings of this study are available on request from the corresponding author.
